# Maximal Fat Oxidation Testing in Gilbert’s Syndrome: Plasma Lipid-Mobilization Signatures from Untargeted Metabolomics

**DOI:** 10.1186/s40798-026-01147-1

**Published:** 2026-11-13

**Authors:** Tamara Christina Stelzer, Hannah Spahits, Anna Maria Kripp, Karin Ortmayr, Lina Maqboul, Agnes Draxler, Claudia Anna Hana, Daniel König, Andrew Cameron Bulmer, Karl-Heinz Wagner

**Affiliations:** 1 Department of Nutritional Sciences, University of Vienna, Josef-Holaubek Platz 2, 1090 Vienna, Austria; 2 Vienna Doctoral School for Pharmaceutical, Nutritional and Sport Sciences (PhaNuSpo), University of Vienna, Vienna, Austria; 3 Department of Sport Science, Centre for Sport Science and University Sports, University of Vienna, Vienna, Austria; 4 Department of Health Sciences, FH Campus Wien, University of Applied Sciences, Vienna, Austria; 5 Department of Pharmaceutical Sciences, Division of Pharmacognosy, University of Vienna, Vienna, Austria; 6 Department of Internal Medicine III, Division of Rheumatology, Medical University Vienna, Vienna, Austria; 7 School of Pharmacy and Medical Sciences, Griffith University, Gold Coast, Australia

**Keywords:** Bilirubin, Metabolomics, Fatmax, Maximal fat oxidation, Metabolic health

## Abstract

**Background:**

Gilbert’s syndrome (GS), defined by an unconjugated hyperbilirubinemia, has been linked to favorable metabolic traits, including greater fat oxidation and distinct metabolite profiles. Whether GS confers a different acute plasma metabolomic response to a FatMax test remains unknown.

**Methods:**

A human age- and sex-matched case–control (GS vs normobilirubinemic controls) study was conducted from March 2023 to December 2023 at the University of Vienna. After a 12-h-overnight-fast 80 participants completed a FatMax test on a cycle ergometer and from 79 the plasma metabolome was analyzed. Blood samples were collected before (baseline) and immediately after exercise. An untargeted plasma metabolomics analysis was performed via LC–MS.

**Results:**

GS and controls differed significantly in serum bilirubin concentrations (GS: 33.5 ± 13.3; C: 12.2 ± 2.72; *p* < 0.001, in µmol/L). Apart from the expected elevations in the subclass of bilirubin in GS, the global acute metabolomic response to FatMax was broadly similar between groups. Exercise-response was similar in all participants, displaying a fat-oxidation signature post-exercise (increase in several acetylcarnitines (dodecoanylcarnitine, tetradecenoyl carnitine, (10Z,13Z,16Z,19Z)-Docosatetraenoylcarnitine), and several fatty acids (especially unsaturated fatty acids)), with a total of 231 metabolites changing in response to exercise. Individuals with higher maximal fat oxidation displayed larger immediate post-exercise increases in selected metabolites, such as fatty acids.

**Conclusion:**

A submaximal FatMax exercise test elicits a robust plasma signature consistent with fatty-acid mobilization and increased fatty-acid availability for oxidation. However, within the constraints of a 12-h fast and exercise at this workload and duration, GS did not demonstrate a distinct acute metabolomic response beyond elevations in bilirubins.

**Supplementary Information:**

The online version contains supplementary material available at https://doi.org/10.1186/s40798-026-01147-1.

## Key Points

- To the best of our knowledge, this is the first study to investigate the acute metabolomic response to a FatMax test and to characterize the metabolic changes induced by submaximal exercise at maximal fat oxidation.
- Although previously reported differences in the metabolomic profile of individuals with Gilbert’s syndrome could not be fully confirmed, we observed a slight trend towards greater fat oxidation in participants with Gilbert’s syndrome.
- The FatMax test elicited a robust fat oxidation-related metabolomic response across all participants, with only moderate differences between maximal fat oxidation tertiles, supporting the concept of FatMax testing also at the metabolomic level.

**Supplementary Information:**

The online version contains supplementary material available at https://doi.org/10.1186/s40798-026-01147-1.

## Introduction

Gilbert’s syndrome (GS) is a condition typified by moderate hyperbilirubinemia associated with a genetic variation of the UGT1A1 gene. This genetic variation leads to moderately elevated, nontoxic blood concentrations of unconjugated bilirubin (UCB). The global prevalence of GS ranges from 5 to 20%, depending on the region [1]. Historically, bilirubin has been considered a toxic end product of heme catabolism, however, more recent research shows potential protective effects of the molecule, with intriguing effects on cellular metabolism. In several studies, a lower body mass index (BMI) as well as a more favorable blood lipid profile has been repeatedly observed [2–4]. Regarding GS-related metabolic traits, the work of Hana et al. [5] identified a specific lipid profile for participants with GS in an age- and sex-matched case control study. Especially ketone bodies such as acetone, β-hydroxybutyrate, acetoacetic acid, and acetyl carnitine were elevated in GS participants in comparison to matched, healthy controls. These authors therefore suggested greater fat oxidation in GS compared to age and sex-matched controls [5]. Despite this initial evidence, the precise impact of exercise on the metabolome in GS has not yet been comprehensively described.

Exercise is a physiological condition in which metabolic flexibility is required. Metabolic flexibility refers to the ability to respond or adapt to conditional changes in metabolic- or energy demand [6]. During exercise, depending on intensity and duration, changes in energy demand and origin of energy, such as fat or carbohydrates, occurs [6]. During submaximal and aerobic exercise, greater use of fatty acids (FA) is expected, as fat is the largest energy store of the body and slowly oxidized fat results in a high-volume adenosine-triphosphate (ATP) production [7]. In general, fat oxidation rate is an indicator for metabolic health [8]. While impaired fat oxidation is associated with obesity and insulin resistance [8], higher rates of maximal fat oxidation (MFO) are typically observed in leaner, fitter and metabolically healthier people [8], making MFO a relevant physiological marker.

Fat oxidation is measured by indirect calorimetry [9]. MFO and FatMax are determined during a FatMax test, which is a graded exercise test on a cycle ergometer or treadmill. During this test, gas exchange is continuously measured to calculate fat oxidation rates. MFO corresponds to the highest rate of fat oxidation achieved, while FatMax refers to the intensity at which this maximum occurs. Even though FatMax testing has been validated multiple times, to our knowledge, no studies characterized the broader plasma metabolomic response and metabolic shifts during or after a specific FatMax protocol. The focus of recent studies was on estimating total fat oxidation from gas exchange data, however, which specific fatty acids are mobilized or oxidized have not been investigated. A systematic review of exercise metabolomics studies has established that acute aerobic exercise reliably increases circulating free fatty acids, acylcarnitines, monoacylglycerols, and ketone bodies, reflecting enhanced lipolysis and fatty acid oxidation [10]. This signature has since been characterized in further molecular detail, including its temporal dynamics across multiple omics layers [11]. However, whether and how this established signature manifests specifically during a standardized FatMax [11] protocol has not been characterized. The present study therefore aims to characterize this response specifically in the context of a FatMax test. Metabolomics could therefore be an important tool to provide insight into exercise-induced metabolic shifts and help clarify and confirm proposed underlying mechanisms such as fatty acid mobilization and oxidation.

An exploratory metabolomics study was conducted to obtain a more precise picture of substrate utilization during a FatMax test, given that the metabolome can reflect the specific energy substrates used during exercise. Moreover, we investigated whether impaired bilirubin metabolism leads to greater FA oxidation during a FatMax test. Due to earlier findings indicating enhanced FA oxidation in GS individuals [5], we hypothesize that also in this study a greater fat catabolism signature, such as greater increases in acetylcarnitines and ketone bodies, would occur in the GS group.

## Methods

### Study Design and Participants

A metabolic flexibility study was conducted, as previously described in detail [12] which was part of the larger Austrian Science Fund-funded “BiliMetHealth” project. The study design was a human case control study. “Cases” were people with GS and controls were individuals with plasma bilirubin concentrations in the normal range, the groups were age- and sex-matched. The study took place at the Nutrition and Training Laboratory (NuTraLab) of the University of Vienna from March 2023 to December 2023. Participants were recruited from posts on social media and the homepage of the University. Two study appointments were necessary. In the first visit eligibility was assessed by a preliminary examination, which included a medical history interview and a routine blood test. Blood parameters such as total blood count, liver function test, blood glucose, urea, triglycerides, cholesterol, albumin, and C-reactive protein (CRP) were assessed. These analyses were conducted by a certified routine laboratory (SYNLAB, Vienna, Austria). Furthermore, using high-performance liquid chromatography (HPLC), UCB concentrations were assessed as described previously [3, 13]. If UCB concentrations exceeded 17.1 µmol/L and total bilirubin concentrations were above1.2 mg/dL, participants were assigned to the GS group. Besides elevated bilirubin concentrations, another criterion for diagnosing GS is the exclusion of liver diseases. This was done by assessing the concentrations of liver enzymes such as aspartate aminotransferase (AST), gamma-glutamyl transferase (GGT), alanine transaminase (ALT), and lactate dehydrogenase (LDH). Liver diseases were also an exclusion criterion for the control group; therefore, previously described parameters were checked in all participants. Both groups were overall healthy non-smokers, without any acute or chronic metabolic or inflammatory diseases, aged between 18 and 65 years, and had to be moderately physically active (2–3 times per week). Participants with GS were age and sex-matched to normobilirubinemic controls.

The Ethics commission of the University of Vienna (#00905) approved the study. All participants gave their written informed consent, and the study was conducted with approved guidelines by the Declaration of Helsinki. For analysis the data were pseudonymized.

### FatMax Testing

On the second study visit the FatMax test was conducted on a cycle ergometer. The FatMax test consisted of a graded step protocol for non-athletes, as published by Chrzanowski-Smith et al. [14]. The protocol starts either at 30 W or 40 W, depending on the fitness level of the participant. Because physical activity was assessed solely via the International Physical Activity Questionnaire (IPAQ) [15], the participants' current activity concentrations were used to determine the initial power. Participants with a low physical activity level started with 30 W and all the others with 40 W. During the test, heart rate (Bluetooth Smart HF-sensor H10, Polar Electro Austria GmbH, Vienna, Austria) and breath-by-breath gas exchange (Cortex MetaLyzer 3 B, Leipzig, Germany) were continuously measured. Venous blood samples were taken immediately before and immediately after the FatMax test. $\mathrm{V}$O_2_ and respiratory exchange ratio (RER) were averaged over every 4-min step and substrate utilization was calculated with stoichiometric equations [16]. The highest fat oxidation value was than determined as the MFO. Additionally, FatMax was assessed by plotting MFO (g/min) against power output (W) and determining the highest peak. The test was terminated at a RER of 1.00. Therefore, participants did not reach exhaustion and maximal oxygen uptake ($\dot{\mathrm{V}}$O2max) was not assessed.

The testing conditions were kept similar for every participant. Participants fasted 12 h overnight prior to the test and had to follow specific dietary guidelines the day before. These included to eat only moderate amounts of carbohydrates and not to exceed the habitual daily amount of caffeine. Furthermore, participants were instructed to avoid alcohol the day before the test. Compliance with these guidelines was checked with a 24-h-recall. In addition to diet, participants were asked not to engage in strenuous physical activity the day before testing. On the day of testing, participants had to come fasted. The tests all occurred in the morning between 7 and 10 a.m.

### Anthropometric Parameters

The International Standards for Anthropometric Assessments [17] were strictly followed. All assessments required participants to wear light indoor clothing, with measurements taken without shoes on. Anthropometric assessments were performed just before the FatMax test. Body weight was measured to the nearest 0.1 kg (Seca, mBCA 515, Hamburg, Germany) and height was measured to the nearest 0.1 cm with a stadiometer (Seca, freestanding stadiometer with digital display, 274, Hamburg, Germany). Bioelectric impendence analyses (BIA) (Seca, mBCA 515, Hamburg, Germany) was used to assess body composition. To ensure comparability, participants were asked to drink 500 ml of water before the study visit and to empty their bladder before the BIA assessment. Waist-to-hip ratio (WHR) was calculated from the measurement of hip and waist circumference using a tape measure.

### Blood Biochemistry

Venous blood samples were taken before and after the FatMax test. Blood samples were either centrifuged and aliquoted into cryovials as plasma and serum and frozen and stored at – 80 °C for later analysis, or immediately analyzed at the certified laboratory (SYNLAB, Vienna, Austria). For plasma samples either EDTA or heparin was used as an anti-coagulant. Further details regarding sample handling and analysis of blood parameters, as well as genotyping, can be found elsewhere [12].

### Plasma Metabolomics

#### Extraction

Heparinized plasma was used for metabolomics analysis. Samples were assigned to 16 batches. Each batch included both timepoints of a participant and case/control samples were evenly distributed. For analysis, 50 µl plasma was thawed and 200 µl methanol (100%, stored at − 20 °C) was added. Subsequently, the samples were vortexed and placed into an ultrasonic bath (15 min at 10 °C) for further precipitation. After 3 h of incubation at − 20 °C, samples were mixed using an ultrasonic bath (10 min at 10 °C) and centrifuged (13,300 rpm, 20 min at 4 °C). The supernatant was transferred to 1.5 mL vials. For quality control, 5 µL of each sample was pooled into a vial. As an additional quality control sample to monitor potential batch differences, a defined control sample was extracted as part of each batch. Vials were stored at − 80 °C until untargeted metabolomics analysis via liquid chromatography–mass spectrometry (LC–MS).

#### UHPLC–HRMS Analysis

Metabolomics data were acquired using a non-targeted approach on a Sciex X500R LC–QTOFMS system. Metabolites in plasma samples were chromatographically separated on an Acquity Premier BEH C18 column (2.1 × 100 mm, 1.7 µm particle size, Waters, Milford, MA, USA) using a flow rate of 0.35 ml/min and acidified water (mobile phase A, H_2_O with 0.1% formic acid) and acetonitrile (mobile phase B, acetonitrile with 0.1% formic acid and 1% H_2_O) as mobile phase components. The column oven was set to 45 °C. The total analysis time per sample (5 µl injection volume) was 8 min, with the following binary gradient program: 0–0.25 min at 5% B, 0.25–2 min to 20% B, 2–2,75 min to 95% B, 2.75–5 min to 99%, 5–5.75 min at 99%, 5.75–6 min to 5% B and re-equilibration at 5% B until 8 min. The column effluent was connected to the QTOFMS system using an electrospray ionization interface (Sciex Turbo V Ion Source) operated in negative ionization mode at −4000 V spray voltage and 450 °C, with a declustering potential of −70 V and gas flows set to 30 psi curtain gas, 55 psi ion source gas 1, 50 psi ion source gas 2. High-resolution MS/MS analysis was conducted in the mass range from 50 to 1200 m/z in information-dependent acquisition (IDA) mode. IDA was configured for max. 10 candidate ions, an intensity threshold of 25 counts/second, dynamic background subtraction and exclusion of former candidate ions for 3 s. For IDA spectra acquisition, CAD gas was set to 7 psi, collision energy to −20 V, and TOF start and stop masses were 40 and 1000 Da, respectively.

Raw MS/MS data (wiff files) were imported to mzmine (4.7, mzio GmbH, Bremen, Germany) for feature finding (RT tolerances intra-sample 0.04 min, sample-to-sample 0.1 min, noise threshold: 100 counts in MS1, 50 counts in MS2, minimum feature height 500 counts, m/z tolerances scan-to-scan 0.005 m/z, intra-sample 0.0015 m/z and sample-to-sample 0.004 m/z). Features were annotated by spectral matching in mzmine with HMDB, MassBank and LipidBlast spectral databases. Because not all features included measured MS2 spectra, and spectral databases are likewise not complete, we increased the annotation coverage using putative MS1-level annotations. To that end, the feature table was exported from mzmine and imported to MATLAB (2024 a, MathWorks, Natick, MA, USA) for putative annotation using a custom script that compares the measured exact mass to the accurate mass of database-listed metabolites with a mass tolerance of 0.003 m/z. For these MS1-level annotations, the genome-scale model of human metabolism (Recon3D) and the Human Metabolome Database (HMDBv4) were used as databases. The reference masses of known metabolites were calculated from the listed sum formulas, considering deprotonation ([M-H]^−^) and water loss ([M-H-H_2_O]^−^) as the most common form of ionization in negative ion mode.

The annotated dataset was then inspected for batch effects based on the ion intensities of the control sample extracted in each batch of plasma samples. To normalize unavoidable batch offsets, the median ion intensity of all control samples in the full dataset was calculated for each metabolite. Subsequently, the median of control samples was also calculated for each measurement batch, and the ratio of the batch median over the dataset median was used as a multiplicative correction factor to scale all batches to the overall median for each metabolite.

Finally, the annotated feature list with normalized peak area data in each sample was exported in csv file format for further multivariate statistical data analysis.

### Statistical Analysis

#### Sample Size Calculation

Sample size analysis is based on the only metabolomic study so far which showed a difference in acetone levels by 46% between GS and controls [Hana et al., 2021, see protocol]. We assume data to be normally distributed with standard deviation (SD) 20%. If this is the true difference and SD, we will need to study 36 subjects with a power of 80%. The Type I error probability associated with this test is 0.05. To allow for exclusions of 10% of the participants, 40 participants/group will be included in every trial.

#### Data Analysis

Statistical analysis was conducted using R (VersionR-4.5.0). All participants in the GS group were matched to participants within the control group by age (±2 years) and sex. Baseline characteristics of anthropometric, functional, and blood parameters were evaluated with *t*-tests (in case of normal distribution) or Mann–Whitney *U*-tests (if data were not normal distributed), using R. In addition to the groups “GS” and “C”, participants were stratified into "Low", "Medium", and "High" tertiles based on their MFO (g/min/kg/BW), utilizing rank-based splitting to ensure three equally sized groups. Characteristics of the MFO tertiles were evaluated using an ANOVA and Tukey post hoc test in case of normally distributed data and a Kruskal–Wallis ANOVA and Dunn’s post hoc test in case of other data distribution. *P*-values < 0.05 were considered significant. Metabolomics data were log-transformed and standardized for each metabolite relative to its baseline distribution. Specifically, the values at all timepoints were converted to z-scores through subtracting the mean across all participants at baseline and dividing by the corresponding baseline standard deviation. To adjust for multiple testing while accounting for inter-metabolite correlations, the effective number of independent tests (Meff) was calculated. For that, PCA was conducted on the metabolite dataset, and the number of principal components needed to explain 75% of the total variance was defined as Meff (Meff = 39). This value was used to calculate the Meff-adjusted significance threshold (0.05/Meff) and applied to all statistical tests. For timepoint comparisons within each intervention group, unpaired t-tests were conducted with a significance threshold of *α* = 0.05/Meff). A linear mixed effects model (LMM) was fitted to analyze longitudinal changes for each metabolite using packages lme4 [18–20] version 1.1–37, lmerTest version 3.1–3, and emmeans version 1.11.1. Group, time, and the group × time interaction was considered fixed effects and included subject as a random intercept to account for repeated measurements within each participant: value∼Group*Time + *(1∣Subject)*. Pathway analysis was performed on significantly changed metabolites based on their KEGG or HMDB-ID using MetaboAnalyst 6.0 [21] and illustrated with the path view package [22] in R. *P*-values < 0.05 were considered significant and FDR (false discovery rate) was used to correct p-values. In the results and discussion section “t1” refers to the baseline values and “t2” to the post-exercise values.

#### Missing Data

Plasma samples from one participant from the control group at both timepoints could not be analyzed due to technical issues, so the final number for analysis was 79.

## Results

### Baseline Characteristics

The baseline metabolic characteristics of the study population are shown in Table 1. GS individuals had significantly reduced LDL cholesterol concentrations, and a lower, but not significantly reduced BMI. Furthermore, there was a trend to a greater MFO in GS, compared to controls. 5.0% of the controls were TA/TA homozygous, but did not show increased bilirubin concentrations at both (at the preliminary investigation and at the exercise challenge) timepoints.

**Table 1:** Baseline characteristics of the study population

	GS (*n =* 40)	C (*n =* 40)	p-value	Overall (*n =* 80)
Age (years)	36.4 (12.7)	37.0 (12.6)	0.832	36.7 (12.6)
% females	42.5	42.5		42.5
UCB (µmol/L)	31.6 (16.0)	9.91 (3.47)	< 0.001*	20.9 (15.9)
Total bilirubin (µmol/L)	33.5 (13.3)	12.2 (2.72)	< 0.001*	22.8 (14.3)
Height (m)	1.76 (0.09)	1.75 (0.10)	0.746	1.76 (0.10)
Weight (kg)	71.7 (12.9)	75.6 (15.2)	0.223	73.6 (14.1)
BMI (kg/m^2^)	23.0 (2.79)	24.5 (3.75)	0.050*	23.7 (3.37)
FM (%)	24.2 (7.37)	25.9 (9.35)	0.359	25.0 (8.41)
FFM (%)	75.8 (7.37)	73.3 (11.3)	0.247	74.6 (9.58)
WHR	0.80 (0.06)	0.81 (0.07)	0.444	0.80 (0.07)
Glucose (mmol/L)	4.51 (0.60)	4.48 (0.42)	0.817	4.50 (0.50)
Triglycerides (mmol/L)	0.71 [0.38; 4.05]	0.84 [0.37; 2.45]	0.055	0.76 [0.37; 4.06]
Total cholesterol (mmol/L)	4.16 (0.82)	4.45 (0.73)	0.099	4.29 (0.79)
HDL-C (mmol/L)	1.58 (0.33)	1.49 (0.26)	0.164	1.54 (0.30)
LDL-C (mmol/L)	2.24 (0.52)	2.49 (0.54)	0.041*	2.37 (0.54)
IPAQ	2 [1;3]	2 [1;3]	0.133	2 [1; 3]
MFO (g/min/kg body weight)	0.0039 (0.0011)	0.0034 (0.0014)	0.086	0.00366 (0.00126)
FatMax (W/kg body weight)	0.71 [0.40; 2.42]	0.69 [0.33; 2.01]	0.244	0.70 [0.33; 2.42]
Test duration	29.0 (6.79)	27.7 (6.39)	0.394	28.3 (6.58)
Final power output	179 (58.5)	167 (57.3)	0.362	173 (57.9)
Genotype:			< 0.001*	
Homozygote (TA/TA)	82.5%	5.00%		43.8%
Heterozygote (−/TA7)	17.5%	47.5%		32.5%
Wildtype (−/−)	0.00%	47.5%		23.8%

Values are displayed in mean (standard deviation, SD), if normally distributed and in median [minimum; maximum] if not. Significance is marked with a * and in boldface (≤ 0.05)

UCB, unconjugated bilirubin; BMI, body mass index; FM, fat mass; FFM, fat free mass; WHR, waist-to-hip ratio; HDL, high density lipoprotein; LDL, low density lipoprotein; IPAQ, international physical activity questionnaire; MFO, maximal fat oxidation; GS, Gilbert’s syndrome; C, control group

### Differences in Plasma Metabolite Concentrations of GS and Controls Before and After the Fatmax Test

The difference between the GS and the C group at baseline and after the Fatmax test was assessed using a t-test (results are shown in Table 2). Pathway analysis did not reveal any significant results between the groups. Nine metabolites were significantly different at t1, while at t2 there were 11 significantly different metabolites between the GS and C group. The observed differences were confined to metabolites from different lipid classes, as well as bilirubin and biliverdin.

**Table 2:** significant metabolites between groups at both timepoints given as mean differences (GS–C group)

Super class	Class	Subclass	Metabolite	*t*	Mean difference	95% CI (mean difference)	*p*-value	Cohens d [95% CI]
Organoheterocyclic compounds	Tetrapyrroles and derivatives	Bilirubin	Bilirubin	t1	– 1.236	0.89–1.59	< 0.001	1.57 [1.06–2.07]
			Bilirubin	t2	– 1.058	0.70–1.41	< 0.001	1.50 [1.00–1.99]
			Bilirubin	t1	– 1.331	1.00–1.66	< 0.001	1.78 [1.26 − 2.30]
			Bilirubin	t2	– 1.338	1.00–1.68	< 0.001	1.77 [1.24–2.28]
			Biliverdin	t1	– 1.315	0.98–1.65	< 0.001	1.74 [1.22 − 2.26]
			Biliverdin	t2	– 1.324	1.00–1.65	< 0.001	1.79 [1.27–2.31]
			Biliverdin	t1	– 1.309	0.97–1.65	< 0.001	1.73 [1.21 − 2.24]
			Biliverdin	t2	– 1.268	0.95–1.59	< 0.001	1.76 [1.24–2.28]
Lipids and lipid-like molecules	Fatty acyls	Fatty esters	Glutaryl carnitine [M-H-H2O]-	t1	– 0.929	0.53–1.33	< 0.001	1.04 [0.57–1.51]
			Glutaryl carnitine [M-H-H2O]-	t2	– 0.951	0.61–1.29	< 0.001	1.26 [0.77–1.73]
	Prenol lipids	Monoterpenoids	Oxytetracycline	t2	– 0.843	0.40–1.28	< 0.001	0.85 [0.39–1.31]
	Glycerophospholipids	Glycerophosphoinositols	PI(16:0/18:1(11Z))	t2	– 0.857	0.37–1.34	< 0.001	0.78 [0.32–1.23]
			PI(16:0/20:2(11Z,14Z))	t1	– 0.738	0.32–1.15	< 0.001	0.79 [0.33–1.24]
			PI(16:0/20:2(11Z,14Z))	t2	– 0.756	0.36–1.15	< 0.001	0.85 [0.39–1.31]
			PI(16:0/20:4(5Z,8Z,11Z,14Z)) [M-H-H2O]	t1	– 1.066	0.69–1.44	< 0.001	1.25 [0.77–1.73]
			PI(16:0/20:4(5Z,8Z,11Z,14Z)) [M-H-H2O]	t2	– 1.147	0.81–1.49	< 0.001	1.50 [1.00–1.99]
			PI(16:0/22:4(10Z,13Z,16Z,19Z))	t1	– 0.867	0.46–1.27	< 0.001	0.96 [0.49 − 1.42]
			PI(18:0/22:5(4Z,7Z,10Z,13Z,16Z))	t2	– 0.895	0.48–1.31	< 0.001	0.96 [0.49–1.42]
		Glycerophosphoethanolamines	1-Oleoylglycerophosphoethanolamine (Delta 9) [M-H-H2O]-	t1	0.733	-1.15—-0.32	< 0.001	-0.78 [ − 1.24 to − 0.33]
		Glycerophosphocholines	PC(14:0/22:4(7Z,10Z,13Z,16Z))[M-H-H2O]	t2	0.715	-1.10—-0.33	< 0.001	-0.83 [ − 1.29 to − 0.37]

Only metabolites meeting the Meff-adjusted significance threshold are shown

A significant threshold of (alpha = 0.05/Meff) was used

Mean changes are expressed in baseline standard deviation (SD) units of the log-transformed metabolite values

The reference group is the GS group, meaning that negative mean differences indicate a higher concentration of the specific metabolite in the GS group

The seemingly doubled metabolites with same name but different estimates occur when there are two analytical signals (peaks in LC) with different retention times that originate from the exact same mass

This happens particularly often with isomers, but also with other metabolites that have the exact same mass (same molecular formula)

Although the column was able to distinguish that these are different molecules (different chemical properties, structure, polarity, functional groups), they are indistinguishable in the mass dimension used for annotation

### Plasma Metabolite Changes in Response to a Fatmax Test

**Fig. 1:**
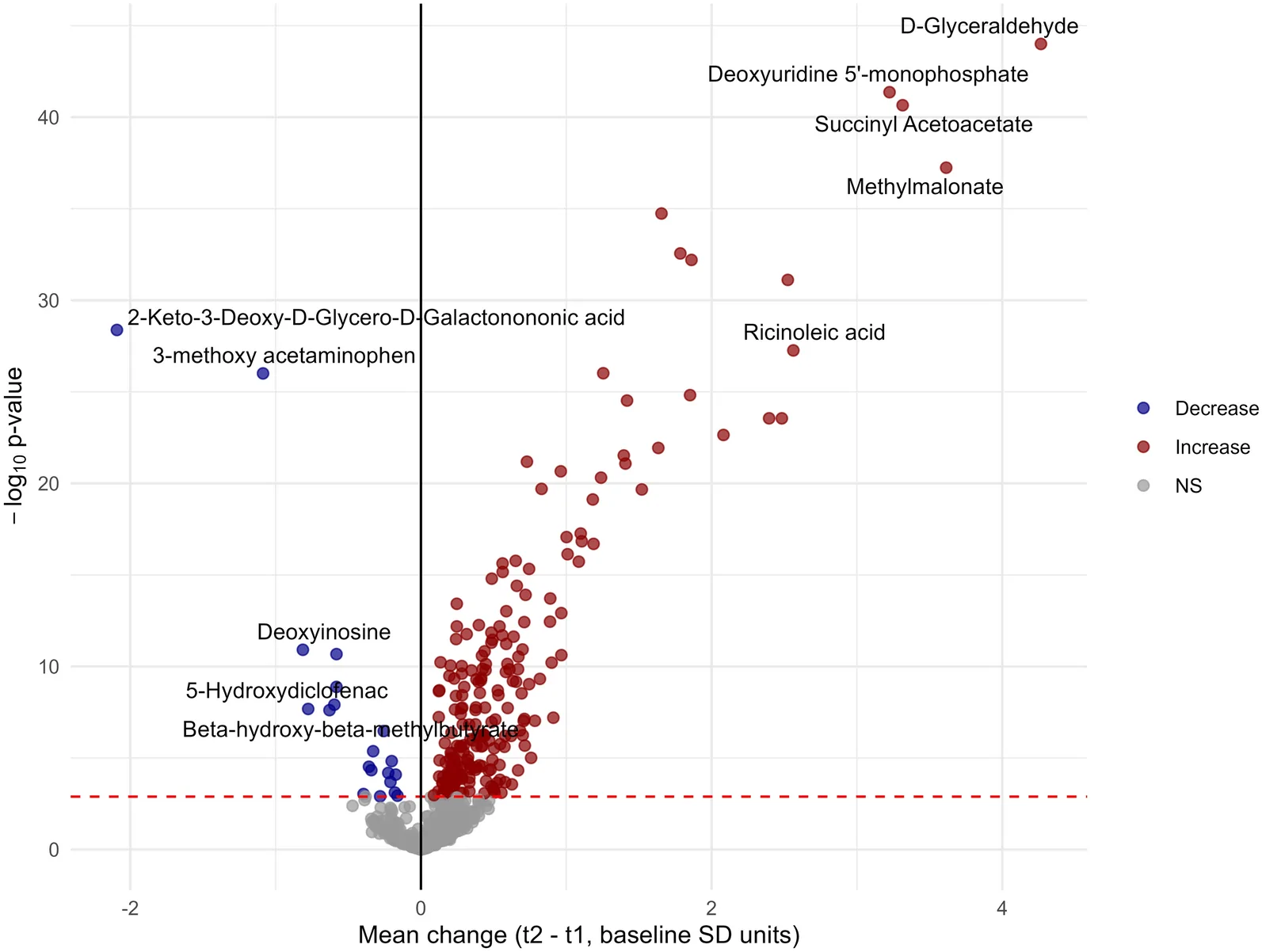
Shows the response of the metabolites changes from t1 to t2 (*n =* 80 paired participants) following the FatMax test. Differences between baseline and after the FatMax test were assessed using paired t-tests. The x-axis shows the mean change (t2 − t1) in baseline standard deviation (SD) units of the log-transformed metabolite values where positive values correspond to an increase and negative values to a decrease from t1 to t2. The y-axis shows −log10(p-value). The dashed horizontal line represents the significance threshold (alpha = 0.05/Meff). The five metabolites showing the largest significant increases and decreases are labeled. NS stands for “not significant”

**Fig. 2:**
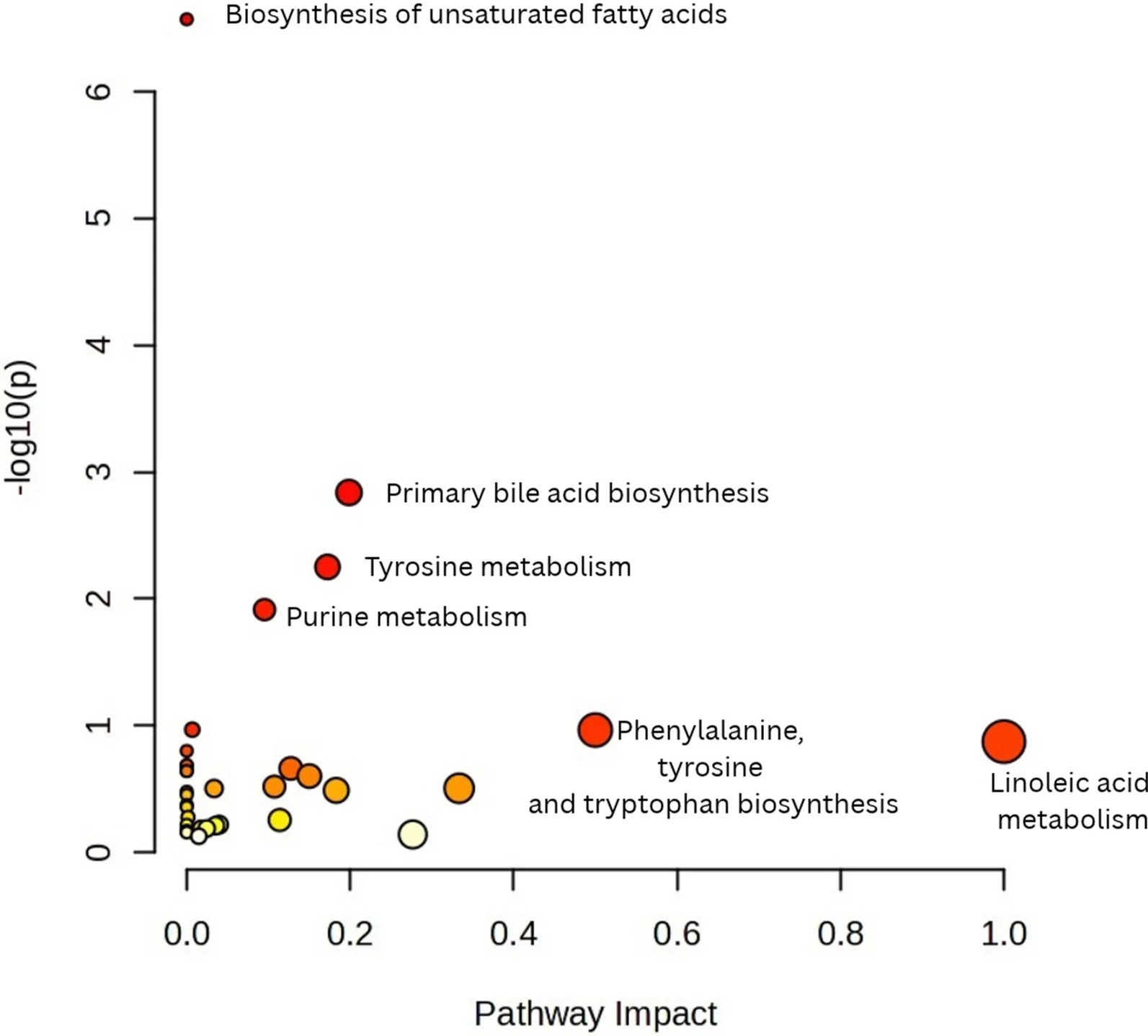
Shows the results of the pathway analysis carried out with MetaboAnalyst [21]. Each bubble corresponds to one pathway. The y-axis shows enrichment significance (− log(p)), and the x-axis shows the pathway impact which is based on pathway topology analysis. Bubble color reflects the enrichment p-value where darker/redder colors correspond to higher significance. Bubble size shows the pathway impact. After FDR correction only “Biosynthesis of unsaturated fatty acids” was significant (*p* < 0.001). Other pathways, not specifically indicated in the diagram were “Nitrogen metabolism”, “Valine, leucine, and isoleucine biosynthesis”, “Phenylalanine metabolism”, “porphyrin metabolism”, “Vitamin B6 metabolism”, “Steroid hormone biosynthesis”, “Biotin metabolism”, “Pyrimidine metabolism”, “alpha-Linolenic acid metabolism”, “Valine, leucine, and isoleucine degradation”, “Tryptophan metabolism”, “Arginine biosynthesis”, “Butanoate metabolism”, “Ubiquinone and other terpenoid-quinone biosynthesis”, TCA cycle, “One carbon pool by folate”, “Alanine, aspartate, and glutamate metabolism”, “Glyoxylate and Dicarboxylate metabolism”, “Glycerophosphatidylinositol (GPI)-anchor biosynthesis”, “Cysteine and methionine metabolism”, “Glycerophospholipid metabolism”, “Arginine and proline metabolism”, “Fatty acid elongation”, “Fatty acid degradation”, “Steroid biosynthesis”, “Arachidonic acid metabolism”, “Fatty acid biosynthesis”

**Fig. 3:**
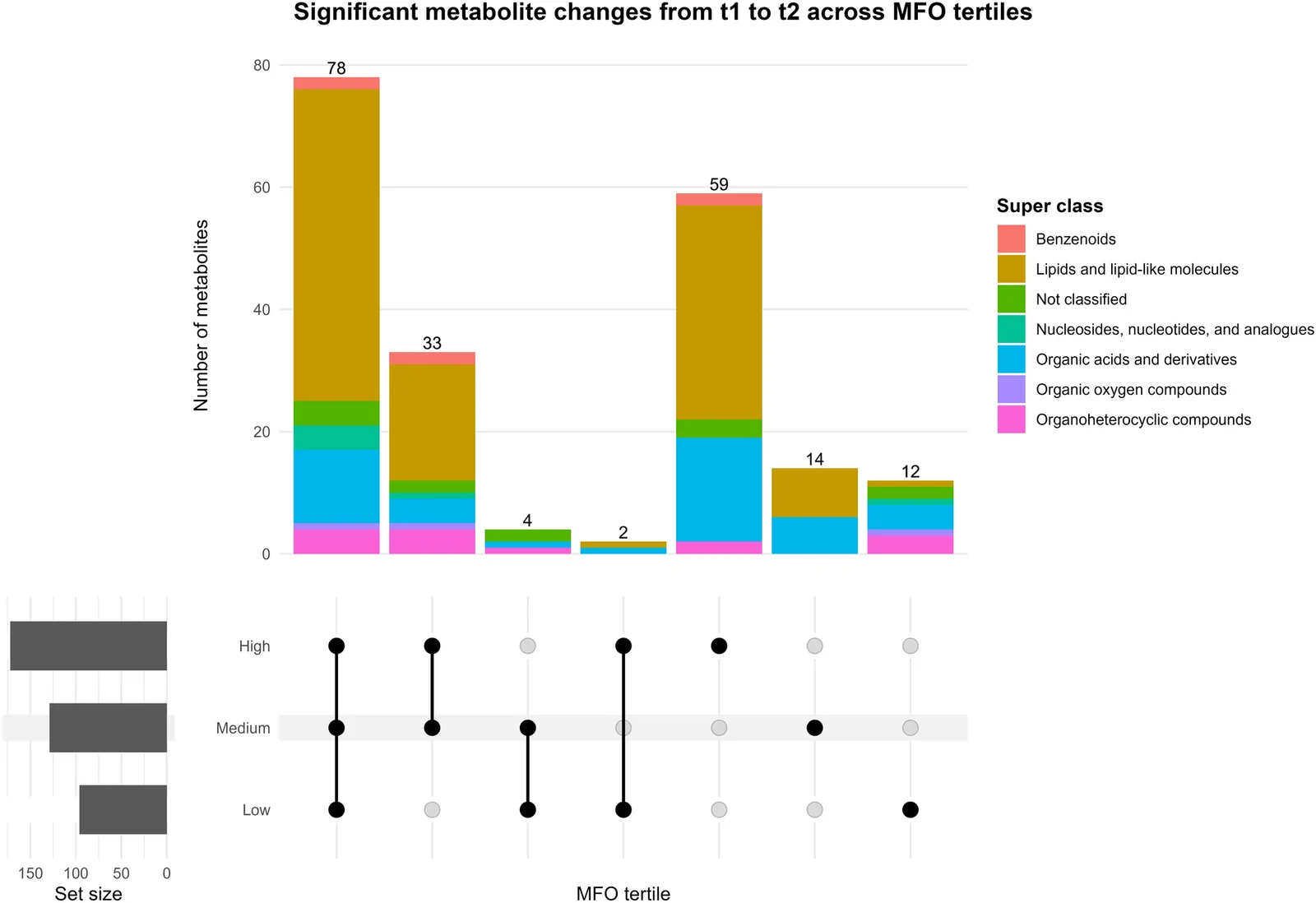
Shows the metabolic change in response to the FatMax test between the MFO tertiles. Metabolites were stratified by super classes

To assess the overall impact of a FatMax test and to obtain a general picture of the intervention effect on the plasma metabolome independent of between-group differences, all participants were analyzed together. A total of 231 metabolites changed in response to the FatMax test, as shown in Fig. 1. Most metabolites increased after the FatMax test. Among those metabolites, mostly lipids and lipid-like molecules increased, except for two glycerophosphoethanolamines. Especially glycerophospholipids, and fatty acyls were highly abundant among the increasing lipid subfamilies. Also in the other classes, namely organic oxygen compounds, organic acids and derivatives, nucleosides, nucleotides and analogues, and benzenoids, most metabolites increased in response to the FatMax test. Details are shown in Appendix A. Furthermore, a pathway analysis was conducted (Fig. 2). One significant pathway was observed, namely “biosynthesis of unsaturated fatty acids”. The significantly changed metabolites within this pathway are illustrated in Fig. 3. In most of the other pathways, only one metabolite from the pathway significantly changed in our data set. However, it should be noted that pathway impact and statistical significance are derived from independent analyses (topological centrality vs. enrichment testing) and are not expected to correlate. Small pathways such as linoleic acid metabolism can yield inflated impact scores from a single centrally positioned hit due to within-pathway normalization, without reaching statistical significance, whereas larger pathways such as biosynthesis of unsaturated fatty acids can achieve high significance through a high hit ratio despite comprising largely peripheral network nodes.

### Stratification into MFO Tertiles

Table 3 shows that MFO tertiles differ significantly in FM, FFM, and IPAQ. The post hoc test revealed a significant difference in FM between the “high” and the “low” MFO tertile (*p =* 0.023). Equally, FFM significantly differed between the “high” and the “low” MFO tertile group (*p =* 0.017). IPAQ was significantly different between “high” and “low” (*p =* 0.011), as well as between “high” and “medium” (*p =* 0.016). Since participants were stratified according to their MFO, MFO significantly differed between all groups (*p* < 0.001).

**Table 3:** Characteristics of the MFO tertiles

	Low	Medium	High	*p*-value
Age (years)	35 [20; 63]	36 [19; 61]	30.5 [19; 57]	0.163
%female	51.9	30.8	46.2	
UCB (µmol/l)	10.5 [4.67; 92.3]	16.2 [6.65; 55.7]	20.7 [4.24; 64.4]	0.204
Total bilirubin (mg/dl)	0.80 [0.50; 5.00]	1.05 [0.50; 3.40]	1.40 [0.50; 3.50]	0.141
Height (m)	1.75 ± 0.10	1.77 ± 0.08	1.76 ± 0.11	0.835
Weight (kg)	70.6 [57.5; 121]	76.7 [51.5; 97.0]	68.2 [47.0; 98.2]	0.325
BMI (kg/m^2^)	23.9 [19.2; 35.8]	23.4 [17.8; 30.2]	22.9 [18.3; 26.3]	0.145
FM (%)	28.0 ± 9.03	25.4 ± 7.63	21.7 ± 7.47	0.030*
FFM (%)	72.0 [55.8; 90.3]	75.0 [59.6; 89.9]	80 [61.5; 88.5]	< 0.001*
WHR	0.79 [0.71; 0.96]	0.81 [0.71; 0.97]	0.78 [0.71; 0.93]	0.278
Glucose (mmol/L)	4.47 (0.35)	4.45 (0.57)	4.52 (0.62)	0.892
Triglycerides (mmol/L)	0.83 [0.40; 4.06]	0.67 [0.37; 2.45]	0.76 [0.40; 2.09]	0.169
Total cholesterol (mmol/L)	4.31 (0.71)	4.21 (0.87)	4.31 (0.73)	0.908
HDL (mmol/L)	1.48 (0.32)	1.48 (0.26)	1.62 (0.31)	0.143
LDL (mmol/L)	2.35 (0.46)	2.33 (0.63)	2.34 (0.53)	0.989
IPAQ	1.00 [1.00; 3.00]	1.50 [1.00; 3.00]	2.00 [1.00; 3.00]	0.007*
MFO (g/min/kg body weight)	0.0025 [1e^-04^; 0.0033]	0.0038 [0.0034; 0.0043]	0.0050 [0.0043; 0.0063]	< 0.001*

Values are displayed in mean (standard deviation, SD), if normally distributed and in median [minimum; maximum] if not. Significance is marked with a * and bold (≤ 0.05)

UCB, unconjugated bilirubin; BMI, body mass index; FM, fat mass; FFM, fat free mass; WHR, waist-to-hip ratio; HDL, high density lipoprotein; LDL, low density lipoprotein; IPAQ, international physical activity questionnaire; MFO, maximal fat oxidation

In the supplementary material (Appendix B1–B3), all metabolites that were significant in the LMM are listed per group and/or interaction in which they were significant. A total of 118 metabolites were significant in the model. Most effects were observed at t2, compared to the reference timepoint t1 in the low group. Eleven metabolites were significantly different in the MFO “medium” group compared to the reference group (MFO “low”) at t1. Three metabolites were significantly different in the MFO “high” group compared to the “low” group at t1. Furthermore, nine metabolites showed a significant Timepoint t2 × Group high interaction, while one metabolite showed a significant Timepoint t2 × Group medium interaction. Most of the metabolites increased over time. Figure 3 shows that most of the significant within-group metabolite changes assessed using t-tests were common across all three MFO tertiles. A smaller proportion of metabolites was specific to MFO “high” or shared between MFO “high” and “medium”. This indicates that the metabolic response was largely shared across tertiles, with some additional changes observed with higher MFO. As the superclass distributions were similar across intersections, these results do not point to class-specific enrichment of shared or tertile-specific signals but show that the MFO groups change in a similar direction with different intensities. Interestingly, participants with GS were more frequently represented in the “high” and “medium” MFO tertiles, whereas control participants were more prevalent in the “low” tertile (), even though this difference in distribution was not significantly different (*p =* 0.443). Further details are shown in the supplementary material (Appendix B1–B3).

## Discussion

This is the first study to investigate plasma metabolomics in people with moderately elevated bilirubin concentrations in response to a FatMax test and also the first study to investigate metabolomics response to a specific FatMax protocol overall. Even though we detected no case–control differences beyond bilirubin/biliverdin, the shared acute metabolomic signature of elevated free fatty acids and acylcarnitines is consistent with enhanced fat oxidation, although plasma concentrations alone cannot distinguish oxidation from altered appearance or clearance.

Previously, only one study investigated the effect of GS on the plasma metabolome and reported that GS had a greater fat oxidation and a specific metabolite profile including acetone and beta-hydroxybutyrate [5], which we were not able to exactly replicate. However, with the trend to a higher MFO and slightly, but not significantly more people in the higher MFO tertiles, we saw a slight trend into the same direction. The group comparison at baseline (t1) and after exercise (t2) indicated a significant difference in bilirubin and biliverdin plasma concentrations. With only seven significantly changed metabolites (namely glutaryl-carnitine and PI(16:0/20:2(11Z,14Z)), which were already significantly different at baseline, oxytetracycline, PI(16:0/18:1(11Z)), PI (16:0/20:4 (5Z,8Z,11Z,14Z)), PI (18:0/22:5 (4Z,7Z,10Z,13Z,16Z)), and PC(14:0/22:4(7Z,10Z,13Z,16Z))) at t2 compared to t1 between groups, the difference between timepoints was rather low, indicating that the metabolic response to the Fatmax test is very similar in both study groups. These group differences in glycerophospholipids after exercise are consistent with altered membrane remodeling in GS relative to C, but the direction is not uniform. Increases in several species in GS coexist with decreases in specific species such as PC(14:0/22:4) and 1-oleoylglycerophosphoethanolamine, suggesting a redistribution among glycerophospholipid classes rather than a global elevation, consistent with the acyl-chain remodeling described by the Lands cycle [23]. Accordingly, we interpret these findings as evidence of differential remodeling dynamics between groups. The elevated metabolite oxytetracycline has no known endogenous function in humans and was most likely misannotated or reflects exogenous/dietary contamination rather than a biologically meaningful GS-associated metabolite. However, it should be noted that all metabolite annotations were putative, and all interpretations rely on this caveat.

Even though Hana et al. reported a significant fat catabolism, the current study and their study had several differences in study design such as a longer fasting period, no exercise challenge and a different assessment method (NMR vs LC/MS), which likely explains the differences in findings.

To isolate the acute effect of a FatMax test on the metabolome, all participants were analyzed together (combining cases and controls). The aim was to metabolically characterize the acute response to the FatMax test by focusing on pathway-level changes in lipid, carbohydrate, and ketone metabolism that are expected by FatMax physiology but have rarely been investigated at the metabolome level. It is well-established that the FatMax testing triggers fat oxidation [9, 14], but little is known on the metabolic response, and which FA actually are oxidized or mobilized. During mild-to-moderate exercise, whole-body fat oxidation increases substantially and peaks at submaximal intensities (FatMax) [24]. However, with increasing exercise intensity, the relative contribution of fat oxidation to ATP generation decreases. Generally, when performing at a submaximal intensity, fat is the primary energy source [25]. MFO therefore occurs during submaximal exercise intensities [26]. Endurance exercise, especially at submaximal intensities, as it is the case for the FatMax test performed in this study, is known to accelerate fat metabolism [27]. The goal of a FatMax test is to identify the MFO, i.e., to force the metabolism to oxidize a maximum amount of fat. The significant accumulation of FAs identified via metabolomic profiling is consistent with systemic lipid mobilization and, tentatively, with enhanced utilization of these substrates during exercise. Mobilization of FA almost certainly exceeded muscular clearance, which is why FA were still elevated at the blood draw immediately after exercise. It is unclear to what extent the oxidized FA originated from adipose tissue as compared to muscle tissue. It might be possible that intramuscular triglyceride (IMTG) oxidation in this study was greater than plasma FFA oxidation [24]. In general, increased FA metabolism during aerobic exercise comes with changes in all forms of lipids (long-chain, medium-chain, PUFAs, amino-FA, acylcarnitines, ketones, monohydroxyls, dicarboxylate, monoacylglycerides, lipid, mediators, membrane lipids) [7], which was also observed in this current study. Moreover, the increase of several acylcarnitines (dodecoanylcarnitine, tetradecenoyl carnitine, (7Z, 10 Z, 13 Z, 16Z)-Docosatetraenoylcarnitine), further support the upregulation of FA oxidation, as a rise in acylcarnitines reflects an increased flux of FA into mitochondria. Moreover, the increase in monoradylglycerols (MG) indicates lipolysis from adipose tissue, as MGs are a breakdown product of triacylglycerol, the storage form of fat in adipocytes.

The significant pathway hit (“Biosynthesis of unsaturated fatty acids”) which was observed during pathway analysis, might not be interpreted as an upregulation of de novo biosynthesis of unsaturated fatty acids, but more as larger proportional mobilization and may also reflect the utilization of unsaturated fatty acids compared to saturated fatty acids. This hypothesis is also supported by the concomitant rise in medium and long-chain acylcarnitines. The selective mobilization of certain FA, especially highly unsaturated FA, was also previously discussed [28].

We hypothesize that the observed changes in glycerophospholipids reflect muscle cell membrane remodeling, potentially involving phospholipase A2 activity. This is based on training-induced LysoPC increases reported in a rodent model [29] and remains to be confirmed in humans.

Consistent with the higher calculated MFO, also the metabolomic profile of individuals with a high or medium MFO indicated a higher fat oxidation (e.g., increased FA and acylcarnitine concentrations) compared to the “low” MFO group. At baseline, acetone and β-hydroxybutyrate, two ketone bodies, were higher than in the “low” MFO group, which indicates a higher whole-body fat oxidation [30] in those two groups compared to the “low” group. This is consistent with earlier findings that individuals with higher fat oxidation possess elevated circulating ketones [30]. Therefore, the MFO and FatMax, originally calculated from the breath-to-breath data could be confirmed by the metabolomics data. Furthermore, the significant Timepoint t2 × Group high interaction for several lipid- and lipid-like molecules underlines the greater fat oxidation in the MFO “high” group. The greater rise in xanthine may reflect a possible greater ATP turnover, as purine intermediates are related to ATP degradation during exercise [31]. This is also reflected in the change in deoxyinosine at t2. Even though these changes were only minor, it was interesting to note that there were slightly more GS participants in, “high” and “medium” MFO groups, than in the “low” group. Also, UCB concentrations in the “high” group were almost twice the concentration as in the “low” group, which further indicates that participants with higher bilirubin have a higher MFO, even if neither the difference in the UCB concentrations, as indicated in Table 3 (*p =* 0.341), nor the distribution between the tertiles, as indicated in Table 4 (*p =* 0.443), was significant. Furthermore, differences in FM, FFM, and physical activity in the tertiles underline the higher fat oxidation in the MFO “high” group, as all these factors contribute to a higher MFO [25].

**Table 4:** Contingency table MFO tertiles (*p =* 0.443; Chi-square test)

Group	Low	Medium	High
GS	11	13	11
C	14	13	14

It is well known that exercise induces changes in the inflammatory immune response [32]. Depending on the intensity and duration of exercise, these changes are more pronounced the higher the intensity and the longer the duration. However also acute bouts of moderate exercise may induce inflammatory responses [33]. Eicosanoids, such as prostaglandins, play a key role in the inflammatory response [34, 35]. In response to the FatMax test, several eicosanoids increased, which may represent the activation of an immune response to acute submaximal exercise.

Besides lipids and lipid-like molecules, an additional increase was observed in other organic compounds. On the level of carboxylic acids and derivatives, the increase in biocytin post-exercise may reflect the turnover of biotinylated acetyl-CoA carboxylases such as acetyl-CoA carboxylase 2, which is crucial for the regulation of FA oxidation [36]. The small rise in creatinine after the FatMax test is not necessarily associated with renal dysfunction, but with an already well-documented response to exercise [37]. However, the increase in creatinine after exercise is most likely associated with high-intensity exercise, since it originates from an accelerated metabolic flux through creatine kinase reactions and an increased phosphocreatine turnover [38]. As already stated, a FatMax test is a submaximal test, where fat is used as a primary fuel. However, the study participants cycled until an RER of 1.00 was reached, which may be more demanding for some of them and may have led to metabolic shifts. Moreover, even if the increase in creatinine is significant, the increase was rather low. Methylmalonate and oxalosuccinate, two metabolites related to the tricarboxylic acid (TCA) cycle were also elevated post-exercise. TCA cycle intermediates are elevated immediately after acute submaximal cycling [39]. However, in that study, participants cycled for 30 min at 10% beyond their first ventilatory threshold, which was a higher intensity and duration than in the FatMax test here. Therefore, even though some minor changes in TCA cycle-related metabolites were observed, the exercise intensity was likely not sufficient to create pronounced changes in energy metabolism.

## Strengths and Limitations

A notable strength of this study is its totally novel study design, since it is the first to measure plasma metabolomics in response to an acute FatMax test. Therefore, we were able to give new insights into the method from a different, metabolomics, perspective.

In this study, we focused solely on changes in the plasma metabolome. Plasma metabolite concentrations, however, do not directly reflect skeletal muscle metabolism. Muscle biopsies would be needed to directly investigate metabolic processes occurring during a FatMax test. Therefore, we can only hypothesize whether the specific FAs measured in plasma were also being oxidized by skeletal muscle. A further asset would have been to have another/some other blood draw/s some hours after the exercise challenge, to better capture post-exercise FA changes. Moreover, we did not measure hemoglobin and hematocrit, which is why we were not able to calculate plasma volume changes and or adjust for them. Furthermore, results might not display the overall population, as the sample included 50% of GS and 50% controls and GS is only prevalent 5–20% in the general population. Furthermore, it should be emphasized that all metabolite annotations in this study are putative, and the interpretations presented here should be considered in light of this limitation.

## Conclusion

These are the first data to document the acute metabolomic response to a FatMax test. Even though earlier findings that people with GS have a unique metabolomic profile could not fully be confirmed, a slight trend toward a metabolomic signature consistent with higher fat oxidation was shown and new findings about the metabolomic response on a FatMax test in general were made. Interestingly, the maximal fat oxidation capacity does not appear to have a strong impact on the plasma metabolome, since group differences between the tertiles of MFO were moderate. However, the FatMax test was associated with a metabolomic signature consistent with greater fat oxidation in every individual. Overall, it can be concluded that a submaximal FatMax test elicited a robust and expected plasma signature consistent with fat oxidation across all participants, without being significantly different between GS subjects and controls and that a submaximal FatMax bout elicits a robust metabolomic response, although this study cannot determine whether this signature is specific to the FatMax intensity or would be observed across a broader range of submaximal exercise intensities.

Beyond the information obtained from indirect calorimetry, metabolomic profiling during a FatMax test may provide additional insight into the metabolic pathways underlying individual responses to exercise, capturing concurrent changes in lipid, carbohydrate, and amino acid metabolism that respiratory measurements alone cannot resolve. This could contribute to a more detailed phenotyping of metabolic flexibility, for example by distinguishing individuals with a similar MFO but divergent underlying metabolomic responses.

## Supplementary Information

Additional file 1.

Additional file 2.

Additional file 3.

Additional file 4.

Additional file 5.

## Data Availability

Raw MS data are uploaded here: https://phaidra.univie.ac.at/detail/o:2350469. Other data that were used for analysis can be obtained upon request from the corresponding author.

## Abbreviations

ALT: Alanine transaminase
AST: Aminotransferase
ATP: Adenosine triphosphate
BIA: Bioelectric impendence analysis
BMI: Body mass index
CRP: C-reactive protein
FA: Fatty acid
FDR: False discovery rate
FFM: Fat free mass
FM: Fat mass
GS: Gilbert’s syndrome
HDL: High density lipoprotein
HMDB: Human metabolic database
HPLC: High-performance liquid chromatography
IDA: Information-dependent acquisition
IMTG: Intramuscular triglycerides
IPAQ: International Physical Activity Questionnaire
KEGG: Kyoto Encyclopedia of Genes and Genomes
LC/MS: Liquid chromatography/mass spectrometry
LDH: Lactate dehydrogenase
LDL: Low density lipoprotein
MFO: Maximal fat oxidation
MG: Monoradylglycerols
PCA: Principal component analysis
RER: Respiratory exchange ratio
RT: Retention time
TCA: Tricarboxylic acid cycle
TG: Triglycerides
UCB: Unconjugated bilirubin
WHR: Waist-to-hip ratio

## References

[1] Goluch Z, Wierzbicka-Rucińska A, Książek E. Nutrition in Gilbert’s syndrome-a systematic review of clinical trials according to the PRISMA statement. Nutrients. 2024. 10.3390/nu16142247. PMC11280271 39064690

[2] Mölzer C, Wallner M, Kern C, Tosevska A, Schwarz U, Zadnikar R, et al. Features of an altered AMPK metabolic pathway in Gilbert’s syndrome, and its role in metabolic health. Sci Rep. 2016;6:30051. 10.1038/srep30051. PMC4956769 27444220

[3] Schoissengeier V, Maqboul L, Weber D, Grune T, Bürkle A, Moreno-Villaneuva M, et al. Association between bilirubin and biomarkers of metabolic health and oxidative stress in the MARK-AGE cohort. iScience. 2024;27(7):110234. 10.1016/j.isci.2024.110234. PMC11253506 39021797

[4] Stec DE, John K, Trabbic CJ, Luniwal A, Hankins MW, Baum J, et al. Bilirubin binding to PPARα inhibits lipid accumulation. PLoS ONE. 2016;11(4):e0153427. 10.1371/journal.pone.0153427. PMC4829185 27071062

[5] Hana CA, Tran LV, Mölzer C, Müllner E, Hörmann-Wallner M, Franzke B, et al. Serum metabolomics analysis reveals increased lipid catabolism in mildly hyperbilirubinemic Gilbert’s syndrome individuals. Metabolism. 2021;125:154913. 10.1016/j.metabol.2021.154913. 34653509

[6] Goodpaster BH, Sparks LM. Metabolic flexibility in health and disease. Cell Metab. 2017;25(5):1027–36. 10.1016/j.cmet.2017.04.015. PMC5513193 28467922

[7] Pellegrino JK, Anthony TG, Gillies P, Arent SM. The exercise metabolome: acute aerobic and anaerobic signatures. J Int Soc Sports Nutr. 2022;19(1):603–22. 10.1080/15502783.2022.2115858. PMC9559054 36250148

[8] Maunder E, Plews DJ, Kilding AE. Contextualising maximal fat oxidation during exercise: determinants and normative values. Front Physiol. 2018;9:599. 10.3389/fphys.2018.00599. PMC5974542 29875697

[9] Achten J, Gleeson M, Jeukendrup AE. Determination of the exercise intensity that elicits maximal fat oxidation. Med Sci Sports Exerc. 2002;34(1):92–7. 10.1097/00005768-200201000-00015. 11782653

[10] Schranner D, Kastenmüller G, Schönfelder M, Römisch-Margl W, Wackerhage H. Metabolite concentration changes in humans after a bout of exercise: a systematic review of exercise metabolomics studies. Sports Med Open. 2020;6(1):11. 10.1186/s40798-020-0238-4. PMC7010904 32040782

[11] Contrepois K, Wu S, Moneghetti KJ, Hornburg D, Ahadi S, Tsai MS, et al. Molecular choreography of acute exercise. Cell. 2020;181(5):1112-30.e16. 10.1016/j.cell.2020.04.043. PMC7299174 32470399

[12] Stelzer TC, Kripp AM, Draxler A, Maqboul L, Pfeiffer KT, Bulmer AC, et al. Metabolic flexibility and exercise performance in adults with Gilbert’s syndrome–associated hyperbilirubinemia. Sports Med Open. 2026;12(1):43. 10.1186/s40798-026-01011-2. PMC13083743 41984342

[13] Wallner M, Antl N, Rittmannsberger B, Schreidl S, Najafi K, Müllner E, et al. Anti-genotoxic potential of bilirubin in vivo: damage to DNA in hyperbilirubinemic human and animal models. Cancer Prev Res (Phila). 2013;6(10):1056–63. 10.1158/1940-6207.Capr-13-0125. 23983086

[14] Chrzanowski-Smith OJ, Edinburgh RM, Thomas MP, Haralabidis N, Williams S, Betts JA, et al. The day-to-day reliability of peak fat oxidation and FAT(MAX). Eur J Appl Physiol. 2020;120(8):1745–59. 10.1007/s00421-020-04397-3. PMC7340634 32488584

[15] Craig CL, Marshall AL, Sjöström M, Bauman AE, Booth ML, Ainsworth BE, et al. International physical activity questionnaire: 12-country reliability and validity. Med Sci Sports Exerc. 2003;35(8):1381–95. 10.1249/01.Mss.0000078924.61453.Fb. 12900694

[16] Jeukendrup AE, Wallis GA. Measurement of substrate oxidation during exercise by means of gas exchange measurements. Int J Sports Med. 2005;26(Suppl 1):S28-37. 10.1055/s-2004-830512. 15702454

[17] Stewart A, Marfell-Jones M, Olds T, De Ridder J. International standards for anthropometric assessment. 2011.

[18] Bates D, Mächler M, Bolker B, Walker S. Fitting linear mixed-effects models using lme4. J Stat Softw. 2015;67(1):1–48. 10.18637/jss.v067.i01.

[19] Kuznetsova A, Brockhoff PB, Christensen RHB. lmerTest package: tests in linear mixed effects models. J Stat Softw. 2017;82(13):1–26. 10.18637/jss.v082.i13.

[20] Lenth R. emmeans: Estimated marginal means, aka least-squares means. R package version 1.8.5. 2023. https://CRAN.R-project.org/package=emmeans.

[21] Pang Z, Lu Y, Zhou G, Hui F, Xu L, Viau C, et al. MetaboAnalyst 6.0: towards a unified platform for metabolomics data processing, analysis and interpretation. Nucleic Acids Res. 2024;52(W1):W398-w406. 10.1093/nar/gkae253. PMC11223798 38587201

[22] Luo W, Brouwer C. Pathview: an R/Bioconductor package for pathway-based data integration and visualization. Bioinformatics. 2013;29(14):1830–1. 10.1093/bioinformatics/btt285. PMC3702256 23740750

[23] Ferrara PJ, Verkerke ARP, Maschek JA, Shahtout JL, Siripoksup P, Eshima H, et al. Low lysophosphatidylcholine induces skeletal muscle myopathy that is aggravated by high-fat diet feeding. FASEB J. 2021;35(10):e21867. 10.1096/fj.202101104R. PMC8439548 34499764

[24] Horowitz JF, Klein S. Lipid metabolism during endurance exercise. Am J Clin Nutr. 2000;72(2 Suppl):558s-s563. 10.1093/ajcn/72.2.558S. 10919960

[25] Purdom T, Kravitz L, Dokladny K, Mermier C. Understanding the factors that effect maximal fat oxidation. J Int Soc Sports Nutr. 2018;15:3. 10.1186/s12970-018-0207-1. PMC5766985 29344008

[26] Achten J, Jeukendrup AE. Maximal fat oxidation during exercise in trained men. Int J Sports Med. 2003;24(8):603–8. 10.1055/s-2003-43265. 14598198

[27] Morville T, Sahl RE, Moritz T, Helge JW, Clemmensen C. Plasma metabolome profiling of resistance exercise and endurance exercise in humans. Cell Rep. 2020;33(13):108554. 10.1016/j.celrep.2020.108554. 33378671

[28] Raclot T. Selective mobilization of fatty acids from adipose tissue triacylglycerols. Prog Lipid Res. 2003;42(4):257–88. 10.1016/s0163-7827(02)00066-8. 12689620

[29] Ortlund E, Hou Z, Chen CY, Gaul D, Zhang T, Moore S, et al. Endurance exercise training alters lipidomic profiles of plasma and eight tissues in rats: a MoTrPAC study. Res Sq. 2024. 10.21203/rs.3.rs-5263273/v1.

[30] Pinckaers PJ, Churchward-Venne TA, Bailey D, van Loon LJ. Ketone bodies and exercise performance: the next magic bullet or merely hype? Sports Med. 2017;47(3):383–91. 10.1007/s40279-016-0577-y. PMC5309297 27430501

[31] Zieliński J, Slominska EM, Król-Zielińska M, Krasiński Z, Kusy K. Purine metabolism in sprint- vs endurance-trained athletes aged 20‒90 years. Sci Rep. 2019;9(1):12075. 10.1038/s41598-019-48633-z. PMC6700101 31427706

[32] Thomas DT, DelCimmuto NR, Flack KD, Stec DE, Hinds TD Jr. Reactive oxygen species (ROS) and antioxidants as immunomodulators in exercise: implications for heme oxygenase and bilirubin. Antioxidants (Basel). 2022. 10.3390/antiox11020179. PMC8868548 35204062

[33] Cerqueira É, Marinho DA, Neiva HP, Lourenço O. Inflammatory effects of high and moderate intensity exercise-a systematic review. Front Physiol. 2019;10:1550. 10.3389/fphys.2019.01550. PMC6962351 31992987

[34] Ricciotti E, FitzGerald GA. Prostaglandins and inflammation. Arterioscler Thromb Vasc Biol. 2011;31(5):986–1000. 10.1161/atvbaha.110.207449. PMC3081099 21508345

[35] Capó X, Martorell M, Sureda A, Tur JA, Pons A. Effects of dietary Docosahexaenoic, training and acute exercise on lipid mediators. J Int Soc Sports Nutr. 2016;13:16. 10.1186/s12970-016-0126-y. PMC4820969 27051354

[36] Zempleni J, Wijeratne SS, Hassan YI. Biotin. BioFactors. 2009;35(1):36–46. 10.1002/biof.8. PMC4757853 19319844

[37] Liu Q, Celis-Morales C, Lees J, Mark P, Welsh P. Effect of exercise on kidney-relevant biomarkers in the general population: a systematic review and meta-analysis. BMJ Open. 2025;15(1):e093017. 10.1136/bmjopen-2024-093017. PMC11749449 39779275

[38] Ávila M, Mora Sánchez MG, Bernal Amador AS, Paniagua R. The metabolism of creatinine and its usefulness to evaluate kidney function and body composition in clinical practice. Biomolecules. 2025. 10.3390/biom15010041. PMC11764249 39858438

[39] Nemkov T, Skinner SC, Nader E, Stefanoni D, Robert M, Cendali F, et al. Acute cycling exercise induces changes in red blood cell deformability and membrane lipid remodeling. Int J Mol Sci. 2021. 10.3390/ijms22020896. PMC7831009 33477427

