# Supplementary material for: Maximal Fat Oxidation Testing in Gilbert’s Syndrome: Plasma Lipid-Mobilization Signatures from Untargeted Metabolomics
Source: Sports Med Open. 2026 Nov 13;12:177. doi: 10.1186/s40798-026-01147-1 (PMC13768531; doi:10.1186/s40798-026-01147-1)
Supplement: Supplementary file 1 [file 40798_2026_1147_MOESM5_ESM.docx]

**Journal:** Sports Medicine – Open

***Title:*** Maximal fat oxidation testing in Gilbert’s syndrome: plasma lipid‑mobilization signatures from untargeted metabolomics

***Authors:*** Tamara Christina Stelzer^ƚ1,2^, Hannah Spahits^ƚ1,2^, Anna Maria Kripp^3^, Karin Ortmayr^5^, Lina Maqboul^1^, Agnes Draxler^4^, Claudia Anna Hana^6^, Daniel König^1,3^, Andrew Cameron Bulmer^7^, Karl-Heinz Wagner^1^

^1^Department of Nutritional Sciences, University of Vienna, Vienna, Austria

^2^ Vienna Doctoral School for Pharmaceutical, Nutritional and Sport Sciences (PhaNuSpo), University of Vienna, Vienna, Austria

^3^ Department of Sport Science, Centre for Sport Science and University Sports, University of Vienna, Vienna, Austria.

^4^ Department of Health Sciences, FH Campus Wien, University of Applied Sciences, Vienna, Austria

^5^ Department of Pharmaceutical Sciences, Division of Pharmacognosy, University of Vienna, Vienna, Austria

^6^Department of Internal Medicine III, Division of Rheumatology, Medical University Vienna, Vienna, Austria

^7^School of Pharmacy and Medical Sciences, Griffith University, Gold Coast, Australia

^ƚ^ These authors equally contributed to the work

Table 1 Peak heart rate and fat oxidation per stage. Fat oxidation is given in g/min per stage and group. Negative values originate from calculation with stoichiometric formula, however physiologically, no negative values are possible.

|  | **GS** | **C** | **p-value** | **All** |
| --- | --- | --- | --- | --- |
| Peak HR | 172 [133; 208] | 167 [124; 198] | 0.086 | 170 [124; 208] |
| Fat oxidation 30 W (g/min) | 0.24 (0.04) | 0.21 (0.11) | 0.294 | 0.22 (0.09) |
| Fat oxidation 40 W (g/min) | 0.24 [0.09; 0.4] | 0.23 [-0.03; 0.34] | 0.343 | 0.24 [-0.03; 0.4] |
| Fat oxidation 65 W (g/min) | 0.24 (0.10) | 0.21 (0.10) | 0.207 | 0.22 (0.10) |
| Fat oxidation 90 W (g/min) | 0.23 [-0.12; 0.42] | 0.18 [-0.07; 0.45] | 0.214 | 0.19 [-0.12; 0.45] |
| Fat oxidation 115 W (g/min) | 0.17 [-0.06; 0.47] | 0.13 [-0.71; 0.41] | 0.211 | 0.15 [-0.71; 0.47] |
| Fat oxidation 140 W (g/min) | 0.1 [-0.24; 0.41] | 0.04 [-0.13; 0.5] | 0.505 | 0.06 [-0.24; 0.5] |
| Fat oxidation 165 W (g/min) | 0 [-2; 0.41] | 0 [-0.22; 0.46] | 0.427 | 0 [-2; 0.46] |
| Fat oxidation 190 W (g/min) | 0 [-0.13; 0.34] | 0 [-0.18; 0.48] | 0.054 | 0 [-0.18; 0.48] |
| Fat oxidation 240 W (g/min) | 0 [-0.19; 0.07] | 0 [-0.24; 0.34] | 0.600 | 0 [-0.24; 0.34] |
| Fat oxidation 290 W (g/min) | 0 [-0.59; 0.31] | 0 [-0.77; 0] | 0.808 | 0 [-0.77; 0.31] |
| Fat oxidation 215 W (g/min) | 0 [0; 0.1] | 0 [-0.18; 0.11] | 0.123 | 0 [-0.18; 0.11] |
| Fat oxidation 265 W (g/min) | 0 [-0.11; 0] | 0 [-0.24; 0] | 0.776 | 0 [-0.24; 0] |
